# Microbial hydrogen economy alleviates colitis by reprogramming colonocyte metabolism and reinforcing intestinal barrier

**DOI:** 10.1080/19490976.2021.2013764

**Published:** 2022-01-13

**Authors:** Li Ge, Jie Qi, Bo Shao, Zhenzhen Ruan, Yueran Ren, Shujing Sui, Xinpei Wu, Xueqiang Sun, Shuman Liu, Sha Li, Changqing Xu, Wengang Song

**Affiliations:** a Shandong Provincial Key Laboratory for Rheumatic Disease and Translational Medicine, The First Affiliated Hospital of Shandong First Medical University & Shandong Provincial Qianfoshan Hospital, Jinan, China; b College of Basic Medical Sciences & Institute of Basic Medical Research, Shandong First Medical University & Shandong Academy of Medical Sciences, Jinan, China; c Department of Gastroenterology, The Affiliated Taishan Hospital of Shandong First Medical University & Shandong Academy of Medical Sciences, Taian, China; d College of Laboratory Animal & Shandong Laboratory Animal Center, Shandong First Medical University & Shandong Academy of Medical Sciences, Jinan, China; e Department of Gastroenterology, The First Affiliated Hospital of Shandong First Medical University & Shandong Provincial Qianfoshan Hospital, Jinan, China

**Keywords:** Microbial hydrogen economy, colitis, hydrogen-rich saline, microbiome, short-chain fatty acids, colonocyte metabolism, intestinal barrier

## Abstract

With the rapid development and high therapeutic efficiency and biosafety of gas-involving theranostics, hydrogen medicine has been particularly outstanding because hydrogen gas (H_2_), a microbial-derived gas, has potent anti-oxidative, anti-apoptotic, and anti-inflammatory activities in many disease models. Studies have suggested that H_2_-enriched saline/water alleviates colitis in murine models; however, the underlying mechanism remains poorly understood. Despite evidence demonstrating the importance of the microbial hydrogen economy, which reflects the balance between H_2_-producing (hydrogenogenic) and H_2_-utilizing (hydrogenotrophic) microbes in maintaining colonic mucosal ecosystems, minimal efforts have been exerted to manipulate relevant H_2_–microbe interactions for colonic health. Consistent with previous studies, we found that administration of hydrogen-rich saline (HS) ameliorated dextran sulfate sodium–induced acute colitis in a mouse model. Furthermore, we demonstrated that HS administration can increase the abundance of intestinal-specific short-chain fatty acid (SCFA)–producing bacteria and SCFA production, thereby activating the intracellular butyrate sensor peroxisome proliferator–activated receptor γ signaling and decreasing the epithelial expression of *Nos2*, consequently promoting the recovery of the colonic anaerobic environment. Our results also indicated that HS administration ameliorated disrupted intestinal barrier functions by modulating specific mucosa-associated mucolytic bacteria, leading to substantial inhibition of opportunistic pathogenic *Escherichia coli* expansion as well as a significant increase in the expression of interepithelial tight junction proteins and a decrease in intestinal barrier permeability in mice with colitis. Exogenous H_2_ reprograms colonocyte metabolism by regulating the H_2_–gut microbiota–SCFAs axis and strengthens the intestinal barrier by modulating specific mucosa-associated mucolytic bacteria, wherein improved microbial hydrogen economy alleviates colitis.

## Introduction

Inflammatory bowel disease (IBD), including Crohn disease (CD) and ulcerative colitis (UC), is a chronic relapsing inflammatory gastrointestinal disorder mainly characterized by lesions of intestinal mucosal ulcers. The current understanding of IBD pathogenesis implicates a complex interaction among host genetics, host immunity, microbiome, and environmental exposure.^1^Gas-involving theranostics have attracted considerable attention in recent years owing to their high therapeutic efficacy and biosafety.^2^ Hydrogen gas (H_2_) is inherently produced during microbial fermentation in the human colon. H_2_ has been shown to have antioxidant properties; in the healthy colon, physiological H_2_ concentrations might protect the mucosa from oxidative insults, whereas an impaired “microbial hydrogen economy” might facilitate inflammation or carcinogenesis. Indeed, hydrogen metabolism, which reflects the balance between H_2_-producing (hydrogenogenic) and H_2_-utilizing (hydrogenotrophic) microbes, has a primary influence on the colonic mucosal ecosystem.^3^ Therefore, an improved understanding of microbial hydrogen metabolism could provide novel strategies to prevent, diagnose, and manage numerous colonic disorders. Several studies have found that exogenous H_2_ treatment can alleviate inflammatory damage in animal models of IBD, but the mechanisms behind it are less clearly understood.^4–6,^ Simultaneously, a causal relationship between “microbiota dysbiosis” and “impaired microbial hydrogen economy” has never been elucidated, and little is known about the interactions between commensal microbiota, H_2_, and the intestinal barrier in IBD. A more detailed understanding of these interactions should provide insight into understanding IBD pathogenesis and direct future clinical research in this area.

Short-chain fatty acids (SCFAs) produced by the intestinal microbiota through anaerobic fermentation of undigested fiber have multiple roles within the human gut.^7^ Energy procurement of the intestinal epithelial cells(IECs) depends on the metabolism of SCFAs, which mainly includes acetate, propionate and butyrate through β-oxidation.^8^ Microbiota derived butyrate increases O_2_ consumption by IECs, reducing its availability in the gut and causing hypoxia. Notably, butyrate promotes the activation of peroxisome proliferator–activated receptor γ (PPAR-γ) in mouse colonocytes which increases mitochondrial activity and fatty acid β-oxidation, a process that consumes O_2_.^9^ SCFAs are also essential for the maintenance of mucosal immunity by fortifying IEC barrier function. In response to SCFAs, intestinal epithelial goblet cells increased the transcription of mucin genes and inoculation of germ-free mice with SCFAs induced goblet cell differentiation and mucus production.^10^ Additionally, SCFAs alter the tight junction(TJ) permeability of IECs.^11^ Collectively, these observations highlight the role of microbial-derived SCFAs in modulating mucosal homeostasis.

In this study, we found that administration of exogenous H_2_ improves the microbial hydrogen economy, which promotes the production of intestinal SCFAs in a DSS-induced mouse colitis model. Increased SCFAs subsequently regulate the metabolic reprogramming of colonocytes to provide an anaerobic environment in the intestinal lumen. Furthermore, exogenous H_2_-modulation of specific mucosa-associated mucolytic bacteria reinforces the mucous layer architecture and integrity of the epithelial barrier. Collectively, the microbial hydrogen economy improved by H_2_ administration promotes recovery of intestinal homeostasis.

## Results

### HS administration alleviates DSS-induced colitis

**Figure 1.**
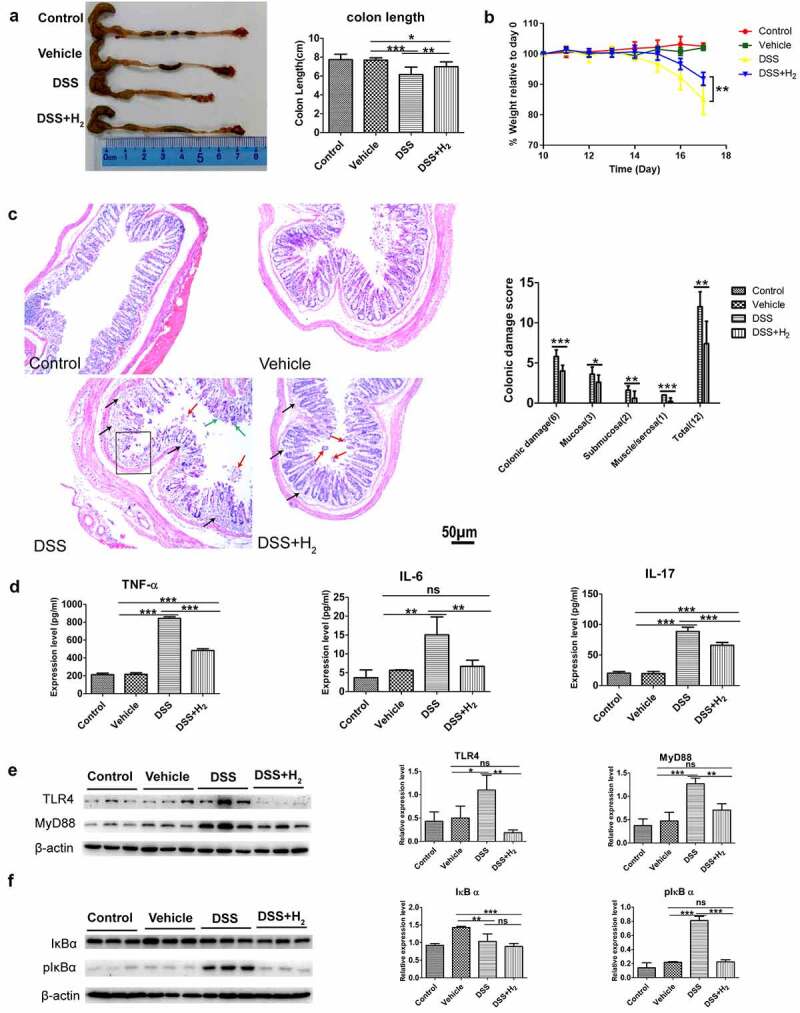
**HS administration alleviates DSS-induced colitis**. (a) Representative colonic photographs and colon length (n = 8). (b) Alteration of body weight from days 10 to 17(n = 8). (c) Representative H&E staining colonic photomicrographs and colonic damage scores. The red arrows denote epithelial shedding, the green arrows denote epithelial hyperplasia, the black arrows indicate inflammatory cell infiltration, and the black box denote crypt loss (Scale bars: 50 μm) (n = 5). (d) The expression levels of pro-inflammatory factors in colonic tissues were analyzed using ELISA (n = 3). (e) Western blotting analyses of MyD88 and TLR4 with β-actin as reference in colon tissue (n = 3). (f) Western blotting analyses of IκB α and phosphorylated IκB α (pIκB α) with β-actin as reference in colon tissue (n = 3). All data show mean ± SD, are representative of two to four independent experiments, and include statistical significance calculated using one-way ANOVA with LSD multiple comparison post hoc test. ns, no significant; * *P* < .05; **P < .01; ***P < .001.

It is increasingly believed that exogenous H_2_ administration can alleviate inflammatory damage in IBD animal models,^4,5,6^ to ascertain the above conclusion, we evaluated the therapeutic efficacy of HS administration against DSS-induced acute colitis. Mice were administered with either normal saline (NS) (DSS group) or HS (DSS+H_2_ group) by intraperitoneal injection throughout the experiment (from days 1 to 17) and exposed to 2.5% (wt/vol) DSS in drinking water (from days 11 to 17). First, HS-administered animals exhibited markedly reduced the susceptibility to DSS-induced colitis, as demonstrated by attenuated colon shortening and weight loss (Figure 1(a,b)). Furthermore, we found that drinking DSS water caused severe pathological processes throughout the distal colon, with extensive epithelial damage and inflammatory infiltrates as well as decreased numbers of colonic crypts, whereas HS treatment protected the colonic epithelium against pathological damage (Figure 1(c)). HS administration also significantly reduced the local levels of pro-inflammatory cytokines, such as tumor necrosis factor α (TNF-α), interleukin (IL)-6, and IL-17 (Figure 1(d)). We also found that HS appreciably inhibited toll-like receptor 4 (TLR-4)/myeloid differentiation factor (MyD88)/nuclear factor κB (NF-κB) signaling pathway by downregulating the protein expression of TLR-4 and MyD88 (Figure 1(e)), which inhibited the phosphorylation of IκBα (Figure 1(f)). Collectively, these results suggest an anti-inflammatory effect of H_2_ on DSS-induced experimental colitis.

**Figure 2.**
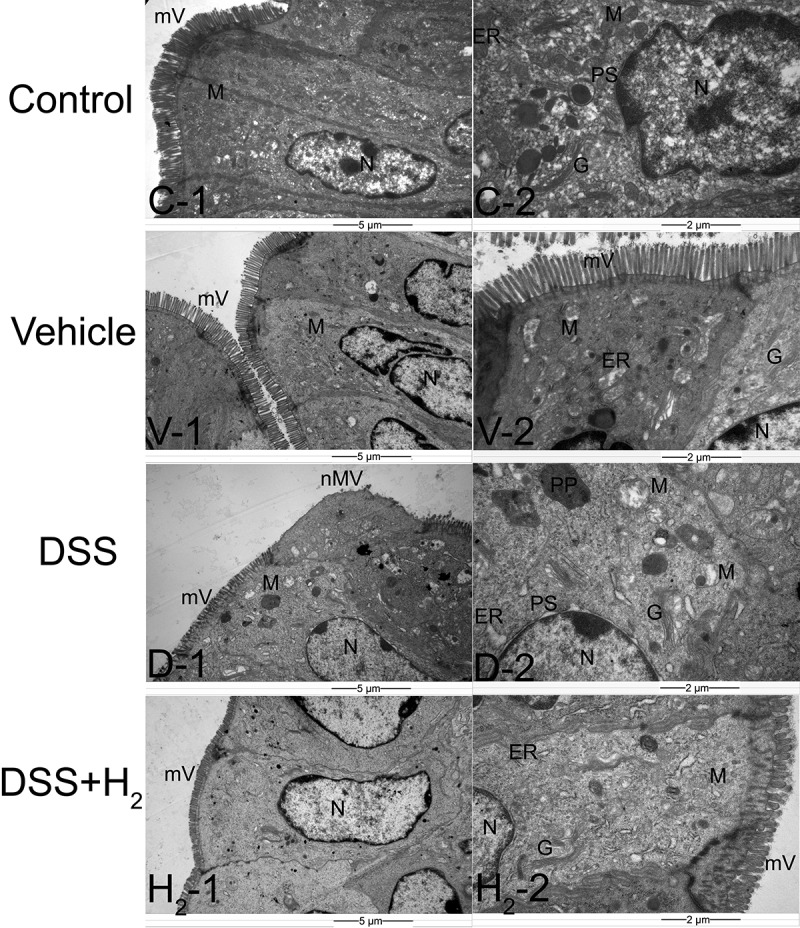
**Representative transmission electron microscopy (TEM) micrographs showing the ultrastructure of colonocytes**. mV: microvillus, nmV: no microvillus, N: nucleus; M: mitochondria, PS: perinuclear spaces, G: Golgi complex, ER: endoplasmic reticulum, PP: pathological products (n = 6). Control group includes C-1, C-2; Vehicle group includes V-1, V-2; DSS group includes D-1, D-2; DSS+H_2_ group includes H_2_-1, H_2_-2. Micrographs are representative of three independent experiments. (Scale bars: the first column, 5 μm; the second columns, 2 μm).

**Figure 3.**
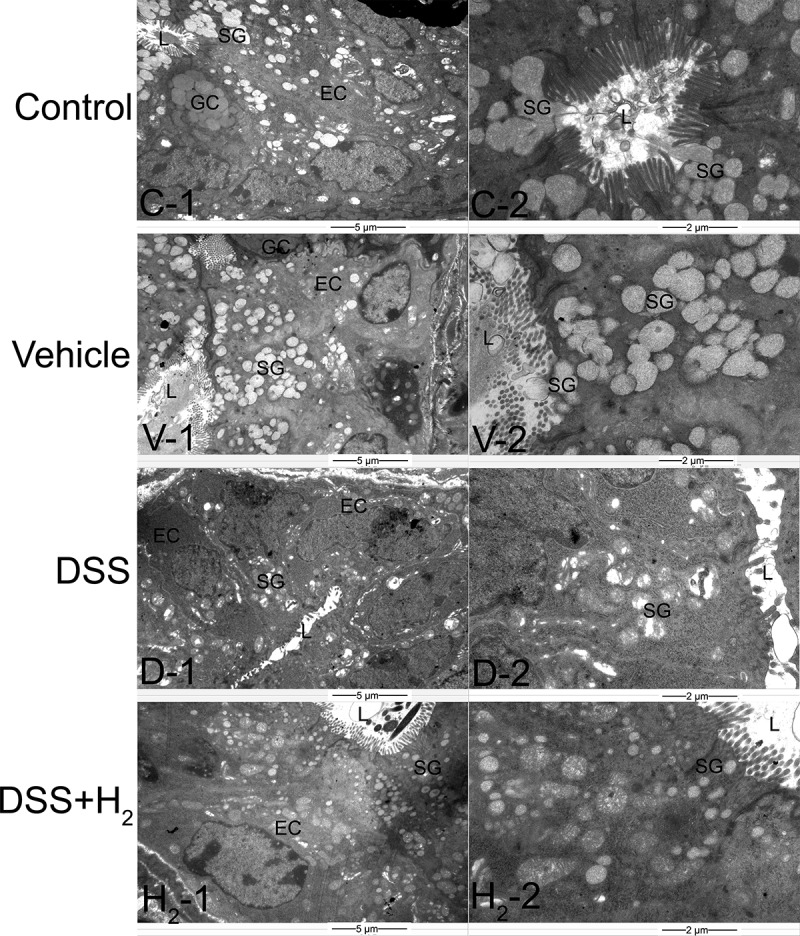
**Representative TEM micrographs showing the ultrastructure of colonic crypts**. EC: epithelial cell, GC: goblet cell, SG: secretory granules, L: lumen of colonic crypt (n = 6). Control group includes C-1, C-2; Vehicle group includes V-1, V-2; DSS group includes D-1, D-2; DSS+H_2_ group includes H_2_-1, H_2_-2. Micrographs are representative of three independent experiments. (Scale bars: the first column, 5 μm; the second column, 2 μm).

Next, we performed ultra-thin sections of colon tissue and observed the ultrastructural changes of colonocytes and colonic crypts among different treatment groups with transmission electron microscopy (TEM) (Figures 2–3). First, dense microvilli were observed on the free surface of colonocytes in the Control group (Figure 2 C-1) and Vehicle group (Figure 2 V-1), while in the DSS group, the microvilli were short and sparse, and even absent in some areas (Figure 2 D-1). Compared with those in the DSS group, the microvilli in the DSS+H_2_ group were almost intact (Figure 2 H_2_-1). Moreover, the ultrastructures of some organelles of the colonocytes, such as mitochondria (M), Golgi apparatus (G), and endoplasmic reticulum (ER), were normal in the Control (Figure 2 C-1, C-2) and Vehicle (Figure 2 V-1, V-2) groups. However, in the DSS group, the damage to the organelles was severe, including edema of the M (Figure 2 D-1, D-2) and G (Figure 2 D-2), and dilation of the ER (Figure 2 D-2). Apparently, HS treatment significantly alleviated the ultrastructural damage to these organelles (Figure 2 H_2_-2). We also observed that the perinuclear space (PS) increased (Figure 2 D-2), and specific pathological products (PPs) appeared in the cytoplasm of the DSS group (Figure 2 D-2). Fortunately, we did not detect the above abnormal phenomenon in the DSS+H_2_ group (Figure 2 H_2_-1, H_2_-2).

The ultrastructures of the epithelial cells (ECs) were intact and had a large number of spherical transparent secretory granules (SG) in the colonic crypt samples of the Control (Figure 3 C-1, C-2) and Vehicle (Figure 3 V-1, V-2) groups. In comparison, in the DSS group, the ultrastructure of the ECs was damaged, and the number of SGs in their cytoplasm was significantly reduced (Figure 3 D-1, D-2). Apparently, H_2_ treatment alleviated the ultrastructural damage of the colonic crypts, as demonstrated by the improved EC ultrastructure and increased SG number (Figure 3 H_2_-1, H_2_-2).

Intriguingly, HS administration exhibited significant efficacy against DSS-induced acute colitis, to some extent, achieving bodyweight recovery, maintaining colon length, and reducing inflammatory damage to the colonic mucosa.

### Modulation of HS administration to the gut microbiome

It is increasingly recognized that the gut microbiome plays a crucial role in human health, and the dysregulated microbiome has been implicated in a number of human diseases, including IBD, obesity, diabetes, cancer, and neurological disorders.^12,13^ Emerging evidence suggests the colonic microbial hydrogen economy is involved in the composition and homeostasis of intestinal microbiota.^3^ Thus, we wanted to determine whether HS administration modulated the gut microbiome in mice with DSS-induced colitis. To illustrate the temporal dynamics of the gut microbiome, we performed a comparative microbiome analysis of feces by performing 16S ribosomal RNA (rRNA) pyrosequencing in the V4 regions. Fecal samples were collected before (at the end of day 10, 10d) and after (at the end of day 17, 17d, before being sacrificed) drinking DSS water. We performed sequencing for a total of 92 fecal samples through 3 independent experiments before and after DSS (9 samples from the control group, 9 samples from the vehicle group, 15 samples from the DSS group, and 13 samples from the DSS+H_2_ group collected before drinking DSS water; 9 samples from the control group, 9 samples from the vehicle group, 15 samples from the DSS group, and 13 samples from the DSS+H_2_ group collected after drinking DSS water).

**Figure 4.**
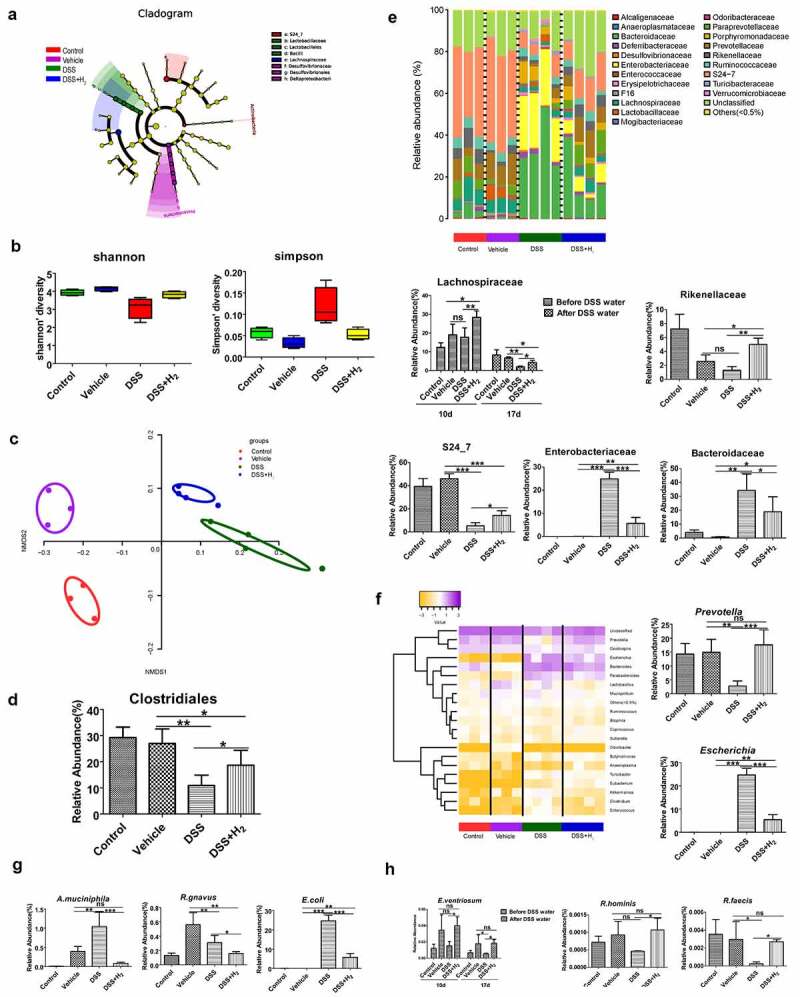
**HS administration induces changes in the composition and diversity of gut microbiota during colitis**. (a) Evolutionary branch diagram of gut microbiome determined with LEfSe analysis before DSS treatment (10 d). (b) α-Diversity estimation of microbial community by observing the Shannon and Simpson indices after DSS treatment (17 d). (c) NMDS plot illustrating the β-diversity of the gut microbiome. Each point represents each mouse, based on unweighted Unifrac distance after DSS treatment (17 d). (d) Relative abundance of Clostridiales at the order level after DSS treatment (17 d). (e) Relative abundance of select taxa respectively at the family level after DSS treatment (17 d) (except for the relative abundance of Lachnospiraceae before (10 d) and after DSS treatment (17 d)). Family-level taxonomy is presented as a percentage of total sequences. (f) Heatmap of the relative abundance of genus-level taxa (rows) for each mouse (columns). The abundance is shown as relative percentage after DSS treatment (17 d). (g) Significantly reduced species in the DSS+H_2_ group through 16s rRNA sequencing after DSS treatment (17 d). (h) Significantly enriched butyrate-producing species in the DSS+H_2_ group through metagenomics sequencing after DSS treatment. Data except Figure 4b are presented as mean ± SD, and include statistical significance calculated with one-way ANOVA with LSD multiple comparison post hoc test. ns, no significant; *P < .05; **P < .01; ***P < .001. For Figure 4b, α-Diversity estimation of microbial community was described with the Median and interquartile range (IQR), and analyzed using the Kruskal-Wallis test. All data are from a representative experiment (n = 3 for Control and Vehicle, n = 4 or 5 for DSS and DSS+H_2_) from three independent experiments.

Given the global alteration of the microbial population, the evolutionary branch diagram of the gut microbiome determined with LEfSe analysis demonstrated no significant differences in taxa between the DSS+H_2_ and vehicle groups before DSS induction (Figure 4(a)). Meanwhile, α diversity analysis, including the Simpson and Shannon indices, indicated that HS administration could improve the richness and diversity of gut microbiota in DSS-induced colitis (Figure 4(b)). The β diversity of the gut microbiome is reflected in the non-metric multidimensional scaling (NMDS) plot among different groups (Figure 4(c)). The gut microbiota obtained from the DSS group was distinct from that of the Control and Vehicle groups. Simultaneously, we found that the microbial community members of the DSS+H_2_ group were slightly different from those of the DSS group. Combining the results of the α diversity and β diversity analyses, we concluded that HS administration could modulate the composition and structure of the gut microbiome in the feces of IBD mice to some extent.

A balanced gut microbiota is characterized by the dominance of obligate anaerobic members of the phyla Firmicutes and Bacteroidetes, whereas an expansion of facultative anaerobic Enterobacteriaceae (phylum Proteobacteria) is a common marker of gut dysbiosis.^14^ Further exploration of the effect of HS administration on microbial communities in IBD mice showed dramatic differences between the DSS and DSS+H_2_ groups at the phylum level in the microbial communities (Figure S1). In the present study, HS administration significantly prevented an increase in the Proteobacteria levels. Interestingly, Bacteroidetes and Firmicutes levels were restored, further supporting the hypothesis that H_2_ modulates the composition of the intestinal flora. At the order level, HS treatment significantly increased the abundance of Clostridiales (phylum Firmicutes) (Figure 4(d)), which are obligate anaerobes that include abundant butyrate producers, specifically Lachnospiraceae and Ruminococcaceae. Notably, at the family level, HS treatment dramatically improved Lachnospiraceae (Figure 4(e)), whereas it had no significant influence on the abundance of Ruminococcaceae in mice with DSS-induced colitis (Figure S2A). It has been shown that Lachnospiraceae could not only inhibits the expansion of *Clostridium difficile* and thus alleviates the development of colitis,^15^ but it can also produces abundant butyrate, which is involved in the energy metabolism of colonocytes.^16,17^ A new study suggested that Lachnospiraceae can produce acetate from H_2_ and carbon dioxide through the acetyl-CoA pathway.^18^ Furthermore, two other beneficial bacteria, Rikenellaceae and S24-7, were more abundant in the DSS+H_2_ group than in the DSS group (Figure 4(e)). Rikenellaceae are both important intestinal hydrogenogenic and hydrogenotrophic microbes^19,20^ and are the predominant acetogens.^20^ S24-7 was more abundant after treatment-induced remission of colitis in mice.^21^ The observations currently limited to murine-based studies suggest that S24-7 is involved in host–microbe interactions that affect gut function and health.^22,23^ In addition, expansion of potentially pathogenic Enterobacteriaceae and Bacteroidaceae was inhibited in the DSS+H_2_ group compared with that in the DSS group (Figure 4(e)). Unfortunately, HS administration slightly decreased the relative abundance of Lactobacillaceae (Figure S2B) and Bifidobacteriaceae (Figure S2C), which are intestinal probiotics before DSS induction. At the genus level, *Prevotella*, which is a butyrate-producing bacterium, was more abundant, whereas the expansion of potentially pathogenic *Escherichia* was inhibited after HS administration (Figure 4(f)). At the species level, there was a decrease in *Akkermansia muciniphila* (*A. muciniphila*) and *Ruminococcus gnavus* (*R. gnavus*) in the DSS+H_2_ group compared in the DSS group (Figure 4(g)). It is known that *A. muciniphila* and *R. gnavus* are mucosa-associated mucolytic bacteria that modulate the thickness of the mucus layer.^24,25^ Overall, this change in commensal bacteria depicts a landscape where HS administration has a mildly beneficial microbiota that favors beneficial species, modulates mucus thickness, and hinders the growth of pathogenic bacteria.

To further characterize the gut metabolic profile and microbiome composition, we collected and analyzed 24 fecal samples (three samples selected from each of the four treatment groups before and after drinking DSS water). Each fecal sample was subjected to metagenomic sequencing, followed by profiling of the microbial community taxonomic composition and functional potential. We found specific taxonomic shifts in the DSS+H_2_ group compared to the DSS group at the species level, including increased abundance of *Eubacterium ventriosum* (*E. ventriosum*) both before and after drinking DSS water, increased abundance of *Roseburia hominis* before drinking DSS water, and increased abundance of *Roseburia faecis* after drinking DSS water (Figure 4(h)). Interestingly, *E. ventriosum, R. faecis* and *R. hominis*, which belong to the *Clostridium coccoides* (or clostridial cluster XIVa) cluster of Firmicutes bacteria, are major butyrate-producing bacteria isolated from the colon.^26^ Unfortunately, perhaps due to the small sample size, there were no significant intergroup differences in the abundance of other butyrate–producing species such as *Eubacterium hallii* (*E. hallii), Eubacterium rectale* (*E. rectale*), and *Butyricicoccus pullicaecorum* (*B. pullicaecorum*), which have hydrogenases that can produce butyrate and H_2_ using acetate and fructose equivalents of fructose, oligofructose, or inulin.^27^ Furthermore, according to differential analysis of gene abundance using metagenomic sequencing, we found in the pathway enrichment analysis of genes based on the Kyoto Encyclopedia of Genes and Genomes (KEGG) database that the DSS+H_2_ and DSS groups have significantly different abundance gene numbers in pathway terms, including starch and sucrose metabolism, pentose and glucuronate interconversions, carbon metabolism, bacterial chemotaxis, etc. (Figure S3).

**Figure 5.**
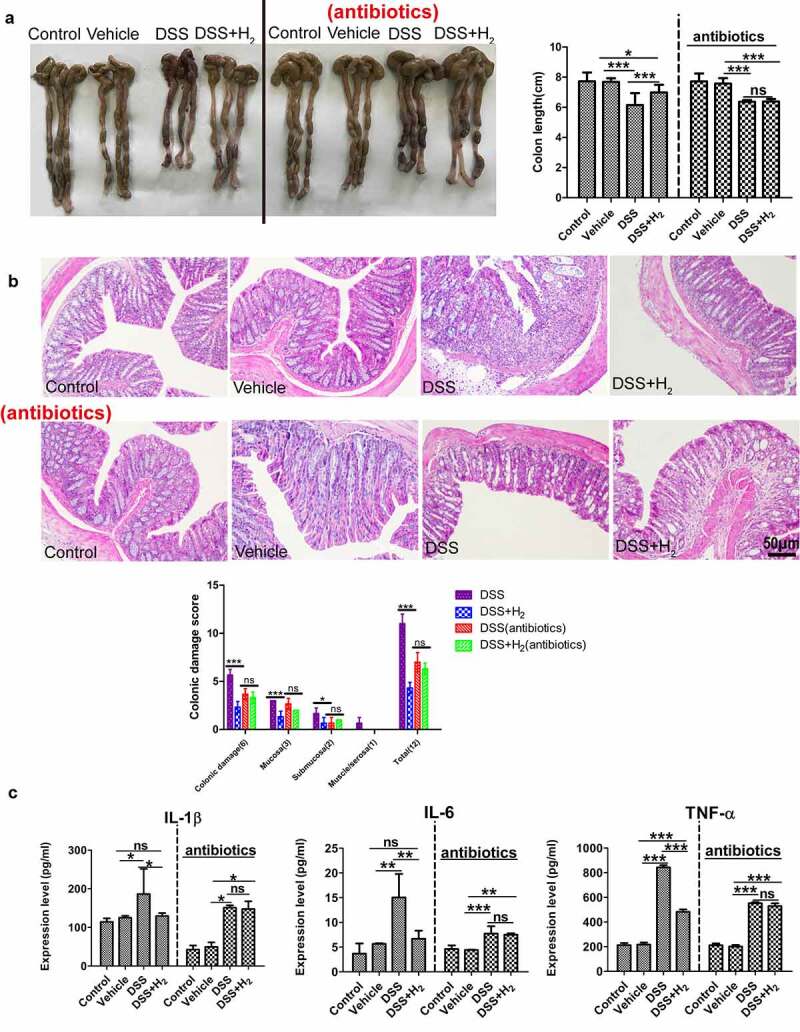
The benefits of HS are partially attributed to modulation of gut microbiome.

Interestingly, pretreatment of DSS mice with broad-spectrum oral antibiotics, known to disrupt gut commensal microbes, resulted in significantly reduced efficacy of HS against DSS-induced colitis (*P* < .05, compared to HS without antibiotics), suggesting that the benefits of HS are partially attributed to the modulation of the gut microbiome (Figure 5). Thus, we ascertained that HS administration could upregulate the abundance of related SCFA-producing bacteria and downregulate the abundance of pathogenic bacteria in mice with colitis, thereby improving the composition of the bacterial community and modulating the gut microbiome.

C57BL/6 mice were pretreated for 5 d with a cocktail of antibiotics (ampicillin, metronidazole, vancomycin, and neomycin) added to the drinking water, and then administered via intraperitoneal injection with HS from days 1 to 17. Mice were provided with water or 2.5% DSS-containing water from days 11 to 17. On day 17, animals were euthanized and colon length (a) (n = 5), colonic damage scores (b) (n = 5), and protein expression of pro-inflammatory factors in colonic tissues (c) (n = 3) were measured. All data are presented as the mean ± SD from a representative experiment (n = 5) are representative of two to four independent experiments. Statistical significance was calculated using one-way ANOVA with LSD multiple comparison post hoc test. ns, no significant; * *P* < .05; **P < .01; ***P < .001.

### Activation of PPAR-γ signaling driven by H_2_-increased SCFAs maintains colonocyte hypoxia during colitis

**Figure 6.**
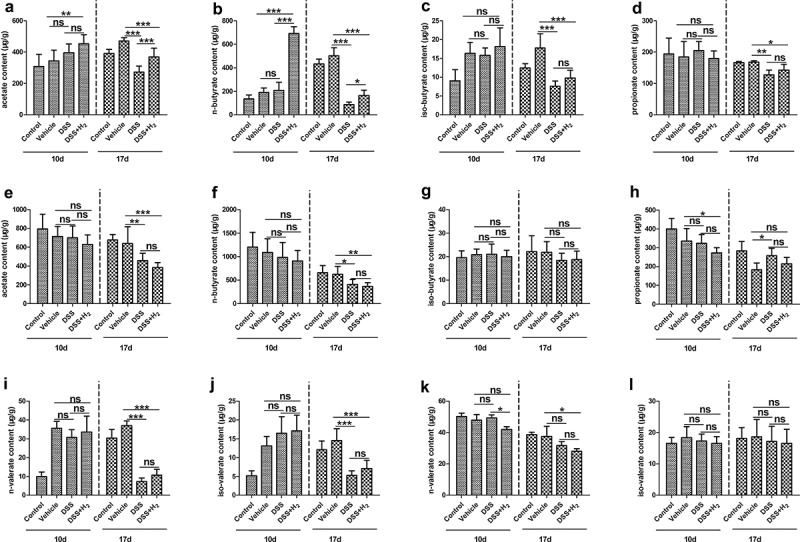
**HS administration affects SCFAs profiles in the cecal and fecal samples**. Different SCFA concentration was determined in fecal samples and cecal contents using GC-MS before (10 d) and after (17 d) drinking DSS water. The concentration of acetate (a), *n*-butyrate (b), *iso*-butyrate (c), propionate (d), *n*-valerate (i) and *iso*-valerate (j) were determined in the fecal samples. The concentration of acetate (e), *n*-butyrate (f), *iso*-butyrate (g), propionate (h), *n*-valerate (k) and *iso*-valerate (l) were determined in the cecal contents (n = 6–8). All data show mean ± SD, are representative of two to four independent experiments, and include statistical significance calculated by one-way ANOVA with LSD multiple comparison post hoc test. ns, no significant; * *P* < .05; **P < .01; ***P < .001.

Although HS administration can increase the abundance of SCFA-producing bacteria, whether HS administration promotes the production of SCFA in the intestine and, if so, whether it has specific effects on SCFA production in different parts of the intestine remains to be determined. To solve this problem, the contents of SCFAs in the cecum contents and feces were determined using gas chromatography combined with mass spectrometry (GC–MS). The results showed that the concentrations of acetate, propionate, and *n*-butyrate in the SCFAs produced by intestinal flora metabolism were higher, whereas those of *iso*-butyrate, *n*-valerate, and *iso*-valerate were much lower than those of the three mentioned above. Compared with NS administration, HS administration markedly increased the production of acetate (Figure 6(a)), *n*-butyrate (Figure 6(b)), and *iso*-butyrate (Figure 6(c)) in fecal samples of mice with DSS-induced colitis; however, there was no significant difference in propionate concentration among the four groups before and after drinking DSS water (Figure 6(d)). For the cecum content samples, although there were no significant differences in the concentrations of acetate (Figure 6(e)) and butyrate (Figure 6(f-g)) between the DSS and DSS+H_2_ groups before and after drinking DSS water, the concentration of propionate in the DSS group was surprisingly significantly higher than that in the DSS+H_2_ group (Figure 6(h)). Since we have only performed microbiome analysis on feces, whether this phenomenon is related to the microbiome changes in cecum contents needs to be further studied. In addition, there were almost no significant differences in the concentrations of valerate between the DSS and DSS+H_2_ groups in either fecal (Figure 6(i-j)) or cecal samples (Figure 6(k–l)). Stool seems to be a promising noninvasive source of bacterial metabolites that are highly sensitive to the inflammatory state.

A recent study suggested that depletion of butyrate-producing microbes by antibiotic treatment reduced epithelial signaling through the intracellular butyrate sensor PPAR-γ. Consequently, elevated colonic epithelial oxygenation promotes a dysbiotic expansion of potentially pathogenic Enterobacteriaceae by increasing the bioavailability of respiratory electron acceptors in the lumen of the colon.^8,16^ To gain further insight into the function of H_2_ in the maintenance of intestinal homeostasis, we next examined the effect of H_2_-regulated microbiota and resulting SCFAs on colonocyte metabolism.

**Figure 7.**
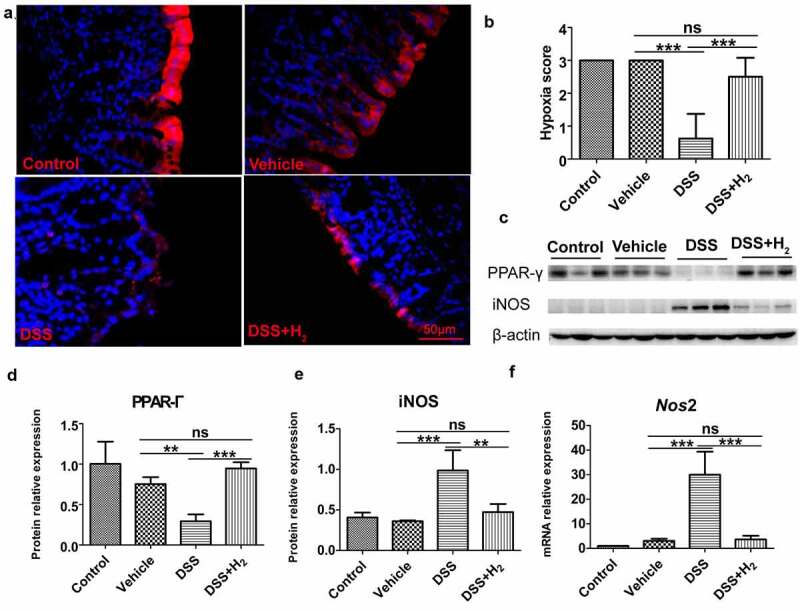
**HS administration maintains colonocyte hypoxia by activating epithelial PPAR- γ signaling during colitis**. (a-b) Immunofluorescent staining for Hypoxyprobe-1(red) and nuclear (blue) in colon tissue and quantification of staining intensities (n = 8). (Scale bars: 50 μm, Magnification: 40x) (c-e) Western blot was used to determine PPAR- γ and iNOS with β-actin as reference in colon tissue (n = 3). (f) Real time RT-PCR analysis of Nos2 (iNOS) transcript levels from colonic epithelia (n = 3). All data show mean ± SD, are representative of two to four independent experiments, and include statistical significance calculated by one-way ANOVA with LSD multiple comparison post hoc test. ns, no significant; * *P* < .05; **P < .01; ***P < .001.

As shown in healthy mice, colonocytes obtain energy through β-oxidation of microbiota-derived butyrate, which consumes a considerable amount of oxygen, thereby rendering the epithelium hypoxic in homeostasis (Figure 7(a-b)), Control and Vehicle). However, in mice with colitis (DSS group), the relative abundance of butyrate-producing microbes including Lachnospiraceae (Figure 4(e)), and *E. ventriosum, R. faecis*, and *R. hominis* (Figure 4(h)), as well as the production of butyrate (Figure 6(b)) decreased significantly, and thus, epithelial PPAR-γ signaling was reduced (Figure 7(c-d)). Nitrate levels would be increased in the colonic lumen because the epithelial expression of *Nos*2, the gene encoding inducible nitric oxide synthase (iNOS), was elevated (figure 7(f)) with a decrease in PPAR-γ signaling (Figure 7(c-d)). Meanwhile, colonocytes switch their energy metabolism to the conversion of glucose into lactate, and metabolic polarization of colonocytes toward anaerobic glycolysis is accompanied by elevated iNOS synthesis (Figure 7(c,e)). In agreement with these data, elevated colonic epithelial oxygenation (Figure 7(a-b), DSS) promoted a dysbiotic expansion of potentially pathogenic Enterobacteriaceae (Figure 4(e)) in mice with colitis. The anaerobic environment of the intestinal cavity is broken. Fortunately, HS administration significantly inhibited the reduction of PPAR-γ signaling (Figure 7(c-d)) and reduced the expression of *Nos*2 (Figure 7(f)) and iNOS (Figure 7(c,e)), thereby inhibiting the concentration of lactate and nitrate and the expansion of pathogenic Enterobacteriaceae in the colonic lumen (Figure 4(e)). Therefore, HS administration activated epithelial PPAR-γ by increasing the abundance of butyrate-producing microbes and the production of butyrate which participates in the metabolic reprogramming of colonocytes, decreases colonocyte oxygenation during colitis, and thus maintains colonocyte hypoxia (Figure 7(a-b), DSS+H_2_), thereby promoting the transition from a dysbiotic community to a homeostatic community in the colonic lumen of mice with colitis.

### HS administration ameliorates disrupted intestinal barrier functions by modulating specific mucosa-associated mucolytic bacteria

**Figure 8.**
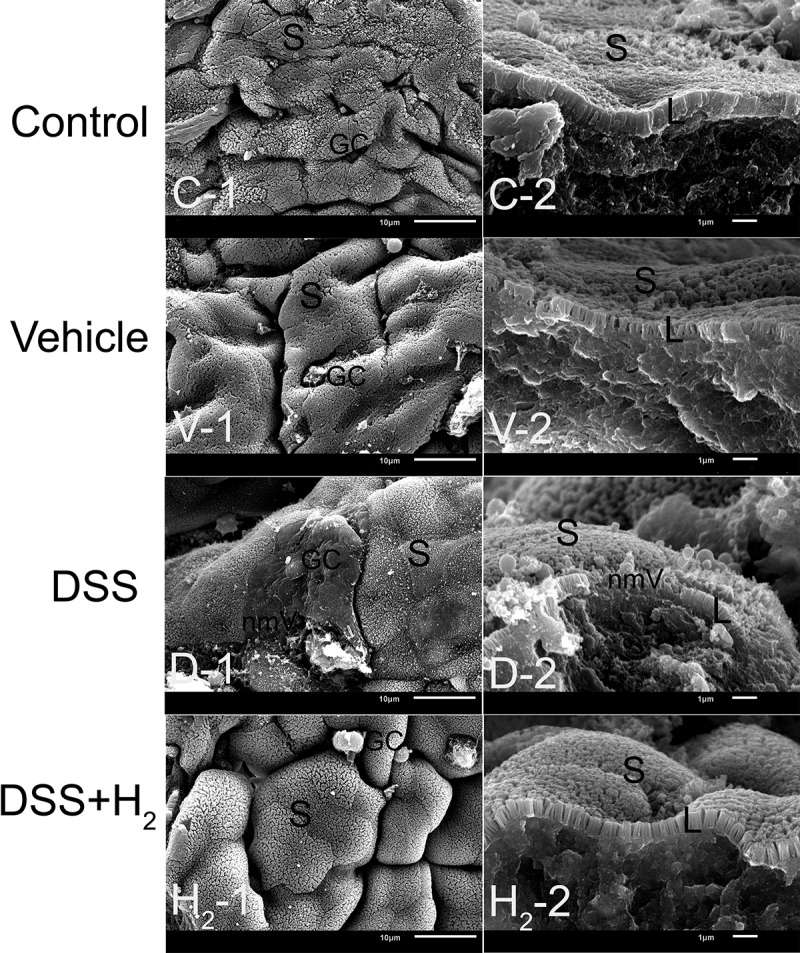
**Representative scanning electron microscopy (SEM) micrographs showing the mucous layer and colonic mucosa**. S: superficial section of colonic mucosa; L: Longitudinal section of microvillus; GC: goblet cell; nmV: no microvillus (n = 6). Control group includes C-1, C-2; Vehicle group includes V-1, V-2; DSS group includes D-1, D-2; DSS+H_2_ group includes H_2_-1, H_2_-2. Micrographs are representative of three independent experiments. (Scale bars: the first column, 10 μm; the second columns, 1 μm).

**Figure 9.**
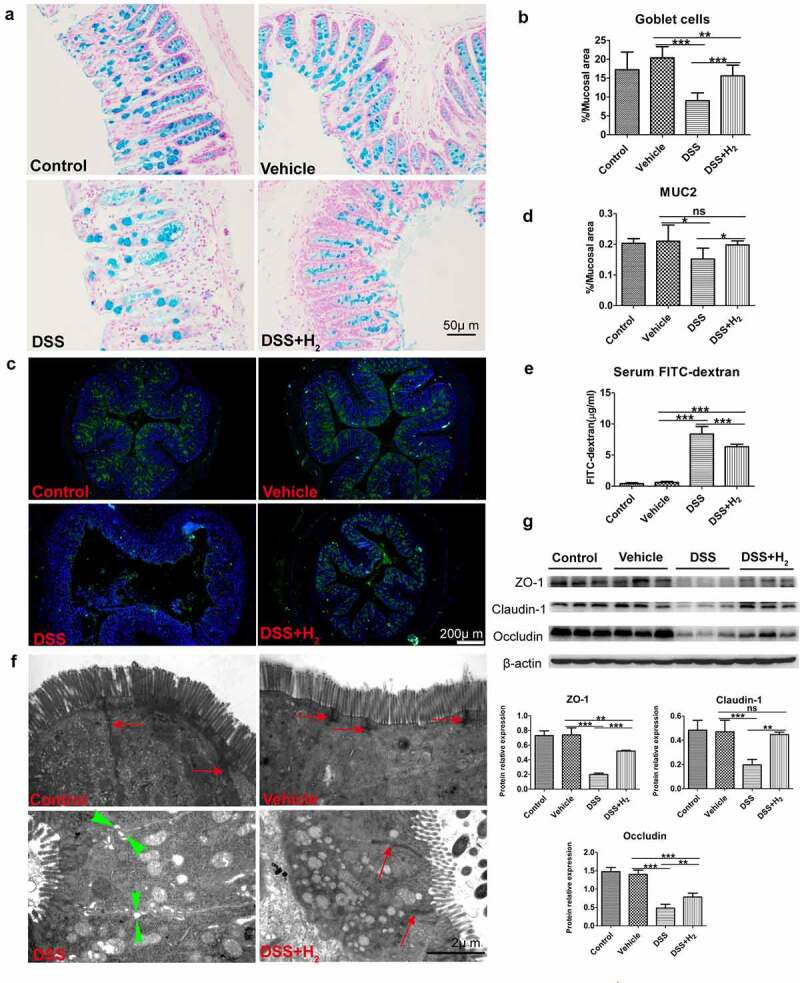
**HS administration reinforces mucous layer architecture and integrity of the epithelial barrier during colitis**. (a-b) Alcian blue staining for goblet cells in colon tissue and quantification of staining intensities (n = 5). (Scale bars: 50 μm). (c-d) Immunofluorescence staining for α-MUC2 (green) and nuclei (blue) and quantification of staining intensities (n = 5). (Scale bars: 200 μm). (e) In vivo epithelial barrier permeability (n = 5). (f) Representative TEM micrographs showing the ultrastructure of colonocytes(Scale bars: 2 μm). Red arrows with shafts delineate tight junction domains and opposing green triangular arrowheads point to intercellular space between two neighbored cells (g) Western blot was used to respectively determine intestinal interepithelial tight junction proteins including Occludin, Claudin-1 and ZO-1 with β-actin as reference in colon tissue (n = 3). All data show mean ± SD, are representative of two to four independent experiments, and include statistical significance calculated by one-way ANOVA with LSD multiple comparison post hoc test. ns, no significant; * *P* < .05; **P < .01; ***P < .001.

Intrigued by these results, we next examined the impact of HS on DSS-inflamed colonic epithelium with disrupted intestinal barrier function. The glycoprotein-rich mucus layer that overlies the gut epithelium is the first line of defense against both commensal microbes and invading pathogens. Goblet cells secrete mucin-2 glycoprotein (MUC2) as a disulfide cross-linked network that expands to form an inner layer, which is tightly adherent to the epithelium and is poorly colonized by commensal bacteria.^28^ Studies have implicated reduced or abnormal mucus production in the development of intestinal inflammation and penetration of commensal bacteria in the inner mucus layer in murine models of colitis and UC patients.^29^ In our study, consistent with the TEM results (Figures 2-3), scanning electron microscopy (SEM) observations showed that, compared with healthy mice (Control [Figure 8, C-1, C-2] and Vehicle groups [Figure 8, V-1, V-2]), which had good mucosal surface structures and dense microvilli on the free surface of colonocytes, the mucosal structure was damaged, as evidenced by the absence of microvilli on some colonocytes in the DSS group (Figure 8, D-1, D-2). In contrast, HS administration significantly inhibited DSS-induced damage to the mucosa (Figure 8, H_2_-1, H_2_-2). The mucus layer is a dynamic barrier that is constantly replenished through the secretory activity of goblet cells.^28^ To further ascertain the effect of H_2_ on mucus production, we performed Alcian blue staining of goblet cells and immunofluorescence staining of MUC2 mucins using α-MUC2 antibody in each mouse. The results revealed a decreased number of goblet cells and MUC2 expression in mice with colitis compared with healthy mice (Figure 9(a-d)), suggesting a weakened ability for mucus production in mice with colitis. Unexpectedly, the decrease in the number of goblet cells and MUC2 expression was alleviated in the DSS+H_2_ group (Figure 9(a-d)).

As mentioned above, the balance of the microbial hydrogen economy maintains homeostasis in the microbiota community.^3^In this context, HS administration ameliorated the decrease in the abundance and diversity of microbiota induced by DSS. It has also been shown that the gut microbiota in the intestinal lumen is in close contact with mucin and has a profound regulatory effect on the mucus layer.^30^ In our study, 16S rRNA pyrosequencing analyses in the V4 regions of fecal samples indicated an abundance of variation of two major mucolytic bacteria, *A. muciniphila* and *R. gnavus*, which significantly decreased the relative abundance of *A. muciniphila* and *R. gnavus* in mice with DSS colitis (Figure 4(g)). Thus, we tested our hypothesis that H_2_ exerts its influence on mucus by regulating the mucosa-associated bacterial composition. It has previously been shown that patients with IBDs have substantially increased abundance of *A. muciniphila*, which is known to promote mucus production and expression of TJ proteins,^31,32^ and *R. gnavus*, which are known for their strong mucolytic activity. Mucolytic bacteria are present in healthy humans, where they are an integral part of the mucosa-associated bacterial consortium in the outer mucus layer, because they are required to degrade MUC2 to release substrate for utilization by other bacteria in the consortium inhabiting this niche.^25^ Herein, we rationalized that H_2_ was involved in regulating mucosa-associated mucolytic bacteria and consequently ameliorated the disruption of intestinal barrier functions.

Additionally, we also observed that the in vivo intestinal barrier permeability and level of opportunistic pathogenic *E. coli* were significantly increased in DSS-fed mice (Figure 9(e) and Figure 4(g)). Scanning electron microscopy showed loss of TJ domains, irregular gaps, and increased distance between adjacent colonocyte cells in DSS-fed mice (Figure 9(f)). Consistent with this, we observed a significant decrease in the expression of interepithelial TJ proteins (Figure 9(g)). Meanwhile, HS-administered animals exhibited markedly reduced susceptibility to disrupted intestinal barrier functions (Figure 4(g) and Figure 9(e-g)).

These results indicated that mucus defects were accompanied by distinct alterations in the microbiota community. Mice with DSS-induced colitis experience dysbiotic gut microbiome, mucus layer deficiency, and increased intestinal barrier permeability. HS administration protects against colonic mucus deterioration by modulating the abundance variation of specific mucosa-associated mucolytic bacteria, thereby ameliorating disrupted intestinal barrier functions and the gut microbiome.

## Discussion

The report that H_2_ could selectively reduce the levels of hydroxyl radicals in vitro and exert therapeutic antioxidant activities in a rat model of middle cerebral artery occlusion was a breakthrough in the field of hydrogen medicine.^33^ Accumulating evidence has shown that H_2_ has potent antioxidative, anti-apoptotic, and anti-inflammatory activities in a wide range of disease models.^34–37^ Of specific interest to the field of gastroenterology is the demonstration that H_2_-enriched water/saline reduced experimental colitis in murine models.^4,5^ Surprisingly, oral lactulose, digested by colonic flora, increased the production of endogenous H_2_, which also had significant anti-inflammatory effects on DSS-induced mouse UC. Although antibiotic treatment can eliminate the anti-inflammatory effect of lactulose, the molecular mechanism by which H_2_ regulates intestinal flora has not been thoroughly investigated.^38^ Recently, in a murine model of sepsis, luminal administration of HS has been shown to prevent intestinal dysbiosis and hyperpermeability. This study proposes a new role for H_2_ in the modulation of the gut microbiome.^39^ Herein, our study demonstrated that HS administration promotes rapid recovery from body weight loss and colonic shortening and alleviates colonic mucosal damage in a murine model of DSS-induced acute colitis. However, in the antibiotic preconditioning group, the remission effect of HS on colitis was not obvious. These results suggest that the intestinal microflora is most likely the key target of H_2_. The pathogenesis of IBD is associated with an imbalance of the gut microbiome,^40,41^ disruption of intestinal barrier functions^42^ and subsequent dysregulated mucosal immune responses.^40,41^ Despite long-standing evidence demonstrating the importance of microbial hydrogen metabolism, minimal efforts have been made to manipulate the H_2_–host–microbe interactions in order to affect colonic health.

H_2_ cycling is crucial for the efficiency of intestinal microbial fermentation. H_2_ is predominantly produced by fermentative bacteria (hydrogenogens) and is thought to be reoxidized primarily by anaerobic respiratory microorganisms (hydrogenotrophs).^43^ More than 70% of microbial genomes in the HMP GI database can synthesize hydrogenases, showing that H_2_ cycling is a dominant mode of energy conservation in the intestinal anaerobic ecosystem.^44^ The predominant mechanism of H_2_ evolution in this ecosystem is through fermentative processes mediated by the Bacteroidetes and Clostridia members of the Firmicutes.^45^ We reported that HS administration restored the dominance of obligate anaerobic members of the phyla Firmicutes and Bacteroidetes, inhibiting the increase of facultative anaerobic Proteobacteria levels and preventing the development of acute colitis in a DSS-induced mouse model. This is in accordance with previous studies reporting that clinical and experimental IBD severity is associated with an increased Proteobacteria/Bacteroidetes ratio.^46^ More importantly, our results clearly point to the importance of H_2_-induced changes in the composition of the gut microbiome and the observed alterations in SCFA profiles. HS administration can specifically increase the abundance of SCFA-producing bacteria and correspondingly promote the production of SCFAs, especially butyrate and acetate. It is speculated that HS administration promotes the metabolic efficiency of the bacterial community to H_2_ by increasing the abundance of specific SCFA-producing bacteria, and consequently promotes the production of SCFAs. Hydrogenotrophic microbes include acetogens, methanogenic archaea, and sulfate-reducing bacteria.^47^ However, a survey showed the key H_2_-oxidizing enzymes responsible for methanogenesis (NiFe groups 3a, 3 c, 4d, and 4e and [Fe]-hydrogenases) and sulfate reduction (NiFe groups 1a and 1b) were either absent or in low abundance in the 100 million sequence reads analyzed. Therefore, the energetically constrained reductive acetogenesis process is the most important hydrogenotrophic pathway.^45^ Rikenellaceae are both important intestinal hydrogenogenic and hydrogenotrophic microbes.^19,20^ The Lachnospiraceae and Rikenellaceae hydrogenotrophic pathways are primarily reductive acetogenesis in the mode of energy conservation in this anaerobic ecosystem.^18,20^ Lachnospiraceae can also convert acetate to butyrate through the acetyl-CoA pathway. Lachnospiraceae can also use H_2_ to produce butyrate in other ways.^26,48,49^ Herein, Lachnospiraceae is also a strong butyrate producer. Meanwhile, we found that HS administration increased the abundance of *Prevotella* (Bacteroidetes), which is also a butyrate-producing bacteria and may reduce the risk of colon cancer.^50^ However, it is unclear whether the three species mentioned above (*E. ventriosum, R. faecis*, and *R. hominis*) and *Prevotella* produce butyrate through the hydrogenotrophic pathway; thus, further Sanger sequencing of the bacterial genome is needed to ascertain the presence of hydrogenase-coding genes and identify the action sites of H_2_.

According to recent reports that other species of clostridial cluster XIVa such as *E. hallii, E. rectale*, and *B. pullicaecorum* have hydrogenases and can produce butyrate and H_2_ (Figure S4); however, in the context of our study, there were no significant differences among the four groups in terms of abundance.^27^ It is speculated that this may be due to the small sample size of metagenomic sequencing. With the increased attention on the biological and pathophysiological significance of microbial hydrogen economy, the discovery of an H_2_–gut microbiota–SCFAs axis may aid in the identification of novel therapeutic interventions targeting the gut microbiome. In addition, despite the different abundances of gene numbers in pathway enrichment between the DSS and DSS+H_2_ groups according to the pathway enrichment analysis based on the KEGG database in metagenomic profiling, gut metabolome – the molecular interface between host and microbiota are less well understood. To address this, we aim to perform untargeted metabolomic and metagenomic profiling of a large number of samples.

Hypoxia in the gut lumen is required to prevent the expansion of facultative anaerobic bacteria, such as pathogenic *Escherichia* and *Salmonella*.^51^ Butyrate has been shown to instruct colonocytes to consume oxygen through the β-oxidation metabolic pathway and consequently protect the host against the expansion of potentially pathogenic bacteria that can lead to IBD.^8,16^ Indeed, a higher abundance of butyrate-producing bacteria in the gut is associated with a lower risk of intestinal inflammation and gut barrier dysfunction.^52^ Our study unequivocally demonstrated that HS administration dramatically increased the abundance of butyrate-producing bacteria Lachnospiraceae, whereby the resulting increase in butyrate activates PPAR-γ in colonocytes, which in turn represses the expression of *Nos2*. Luminal higher oxygen consumption inhibited the proliferation of facultative pathogenic Enterobacteriaceae. Correspondingly, the metabolic polarization of colonocytes toward anaerobic glycolysis was prevented. In this study, H_2_ had a significant impact on metabolic reprogramming to restore colonocyte hypoxia.

MUC2, the major component of the mucus in the colon, is expressed in the intestinal lining in close contact with the gut microflora, keeping the majority of gut bacteria away from epithelial cells and regulating the intestinal microbial habitat.^53,54^ In this study, we observed a decrease in MUC2 protein expression and goblet cell numbers and a thinner mucus layer in mice with DSS-induced colitis. Interestingly, higher intestinal penetrability and increased abundance of potentially pathogenic *E. coli* in fecal samples were observed in mice with DSS-induced colitis. Surprisingly, mice with DSS-induced colitis who were administered HS showed normalized the expression levels of ZO-1, occludin and claudin-1, which are interepithelial TJ-associated proteins that play pivotal roles in gut homeostasis. Furthermore, HS administration prevented systemic exposure to fluorescein isothiocyanate (FITC)–dextran in mice with DSS-induced colitis, demonstrating the restoration of intestinal barrier function. We also found a relatively thickened mucus layer and decreased *E. coli* abundance in the DSS+H_2_ group. Notably, in this study, we detected remarkable blooms of mucosa-associated mucolytic bacteria including *A. muciniphila* and *R. gnavus* in mice with colitis, whereas HS administration significantly inhibited the increase in abundance of both which may also contribute to the thinner mucus layer. The exact mechanisms of the interactions among H_2_, mucolytic bacteria, and the intestinal epithelium remain to be clearly established using a germ-free mouse model, but the inhibition of excessive mucolysis of these two species is believed to play a role in restoring gut barrier integrity during IBD pathogenesis. Furthermore, it has been shown that among the abundant bacterial genera detected in the colon, several strains of *Ruminococcus* spp. produce substantial concentrations of H_2_ in vitro, while also utilizing H_2_ to produce acetate, belonging to reductive acetogens.^55^ Herein, *R. gnavus* bacteria are probably both hydrogenogenic and hydrogenotrophic. Conversely, *A. muciniphila* was clearly shown to produce no detectable levels of H_2_;^56^ as to whether it can use H_2_, further bacterial gene sequencing is needed to determine the presence of hydrogenase-encoding genes. It is speculated that the abnormal increase in *A. muciniphila* and *R. gnavus* is involved in the disrupted microbial H_2_ economy and, consequently, colonic disorders.

**Figure 10.**
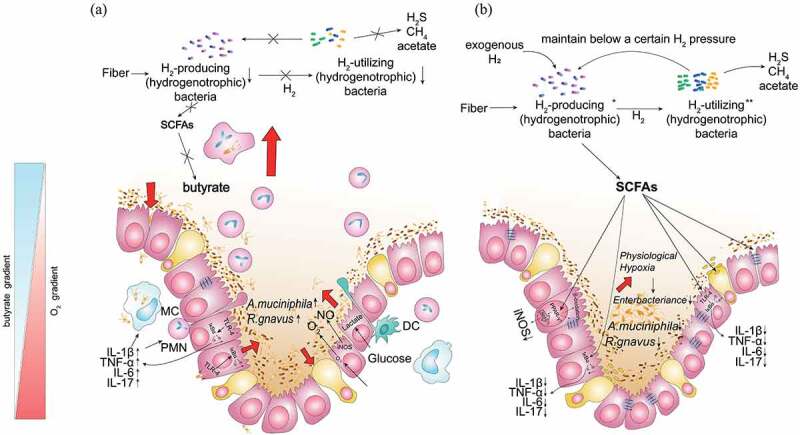
**Microbial hydrogen economy alleviates colitis by reprogramming colonocyte metabolism and reinforcing intestinal barrier**. HS administration can directly target gut microbiota to reprogram colonocyte metabolism and reinforce the intestinal barrier in the inflamed colon.

Our work sheds light on the impact of the microbial hydrogen economy on gut homeostasis in a colitis mouse model. This study demonstrates that HS administration can directly target gut microbiota to reprogram colonocyte metabolism and reinforce the intestinal barrier in inflamed colon while restoring disrupted anaerobic environment and gut microbiome in the intestinal lumen, thus exerting potent ameliorative responses against colitis (Figure 10). Briefly, we developed H_2_ with unique gut microbiome modulatory properties and demonstrated its therapeutic efficacy in a murine model of acute colitis. Further studies are in progress to evaluate the effect of the H_2_–gut microbiota–SCFAs axis in the mouse models lacking PPAR-γ in colonocytes. Undoubtedly, the exact mechanism by which the H_2_–gut microbiota–SCFAs axis regulates colonic mucosal ecosystem still requires further study. Whether H_2_–microbial immunomodulation contributes to gut homeostasis remains to be investigated. Moreover, to further comprehensively understand whether exogenous H_2_ metabolism modulates the balance between hydrogenogens and hydrogenotrophs, future experiments need to address H_2_-induced effects on shifts in microbiota function or activity using bacterial genomic, metagenomic, or metatranscriptomic approaches. In addition, our current studies are limited to the mouse model of DSS-induced acute colitis, and validation in other advanced preclinical models of IBD and IBD patients is needed. Taken together, translating these findings to human microbial hydrogen biology will be essential for understanding the pathogenesis of IBD and will help with the design of future intervention strategies for IBD, where the microbial hydrogen economy plays a central role in determining clinical outcomes.

## Materials and methods

### Nanobubble HS production

Purified H_2_ was dissolved in NS for 20 min using a multifunctional molecular hydrogen generator (NB-B81; Shanghai Nanobubble Technology Co., Ltd, Shanghai, China). The saturated HS was sterilized through filtration and freshly prepared every time to maintain an H_2_ concentration of 3.0 ppm.

### Mice

Male C57BL/6 J mice were purchased from Beijing Vital River Laboratory Animal Technology Co., Ltd., Beijing, China. All animal procedures were reviewed and approved by the Animal Care and Use Committee of the Shandong First Medical University.

### Establishment of a mouse model of DSS-induced colitis

Healthy male C57BL/6 J mice weighing 20–22 g were kept in specific pathogen-free conditions and divided into four groups: control group: the animals received no treatment; vehicle group: each animal received 0.1 mL NS intraperitoneally at 8 a.m. and 4 p.m., respectively, 17 days in a row; DSS group: except for changing normal drinking water to 2.5% (wt/vol) DSS (36–50 kDa; 02160110-CF, MP Biomedicals,) water from days 11 to 17; all other treatments were the same as the vehicle group; DSS+H_2_ group: except for changing NS to HS administration, all other treatments were the same as in the DSS group. All animals were sacrificed at the end of day 17. In some experiments, mice were pretreated by administering a cocktail of antibiotics (ampicillin 1 g/L, vancomycin 0.51 g/L, neomycin 1 g/L; and metronidazole, 1 g/L) in drinking water for 5 days to confirm the depletion of gut microbiome before performing the animal modeling procedure as mentioned above.

For body weight change studies after DSS treatment, the percentage of body weight was determined using the following equation: 100×[(body weight at the indicated day)/(body weight at 10 days)]. The intestinal canal was retrieved from the ileocecum to the anus, and its length was measured. Then, two 0.5-cm pieces in the length of the distal section were used for histological assessment and immunofluorescence staining. The remaining colon tissue samples were split longitudinally, and the contents of the gut were rinsed with phosphate-buffered saline (PBS) and snap-frozen for subsequent molecular biological analysis.

### Histological analysis

The distal colonic tissues were obtained at a distance of 1–2 cm from the anus; these were fixed using 4% paraformaldehyde for 24 h and embedded in paraffin. Slices with a thickness of 3 μm were prepared and stained stained with hematoxylin and eosin. The severity of colonic histological damage was scored in a blinded fashion to prevent observer bias, as previously described.^57^ Briefly, colonic damage was scored as follows: 0, normal; 1, hyperproliferation, irregular crypts, and goblet cell loss; 2, mild to moderate crypt loss (10%–50%); 3, severe crypt loss (50%–90%); 4, complete crypt loss, surface epithelium intact; 5, small- to medium-sized ulcers (<10 crypt widths); 6, large ulcers (≥10 crypt widths). Inflammatory cell infiltration was scored separately for the mucosa (0, normal; 1, mild; 2, moderate; 3, severe), submucosa (0, normal; 1, mild to modest; 2, severe), and muscle/serosa (0, normal; 1, moderate to severe). Scores for epithelial damage and inflammatory cell infiltration were summed, resulting in a total score ranging from 0 to 12.

### Enzyme-Linked Immunosorbent Assay (ELISA)

For the ELISA of TNF-α, IL-1β, IL-6, and IL-17, colons were homogenized in ice-cold lysis buffer (1 mL/0.1 g) containing 1% protease inhibitor cocktail and 1% phosphatase inhibitor cocktail. The lysate was centrifuged (14000 rpm, 4°C) for 10 min, and the supernatants were collected to detect the expression of TNF-α, IL-1β, IL-6, and IL-17. Sandwich enzyme-linked immunosorbent assays were performed with cytokine-specific kits (ELM-TNFα, ELM-IL1b-CL, ELM-IL6-CL, ELM-IL17, RayBiotech, USA) following the manufacturer’s instructions.

### Western blotting assay

Total proteins from colon tissue were prepared using RIPA buffer (Solarbio, China) with a protease inhibitor cocktail (Cwbio, China). For PPAR-γ detection, nuclear proteins from fresh colon tissue were extracted using a nuclear extraction kit (Cwbio, China) according to the manufacturer’s protocol. Protein concentrations were measured using a BCA Protein Assay Kit (Solarbio, China). A total of 40 µg of protein was mixed with sodium dodecyl sulfate loading dye containing 20 mg/mL dithiothreitol reducing agent, boiled for 10 min, separated by sodium dodecyl sulfate–polyacrylamide gel electrophoresis, and wet-transferred to polyvinylidene difluoride membranes (0.45 μm; Millipore). The membranes were blocked for 2.5 h at room temperature with 5% nonfat milk in Tris-buffered saline with 0.1% Tween 20 detergent (TBST) and incubated overnight at 4°C with specific primary antibodies: rabbit anti-TLR4 monoclonal antibody (1:500; 66350-1-1 G, Proteintech), rabbit anti-MyD88 monoclonal antibody (1:1000; #4283), mouse anti-IκB-α monoclonal antibody (1:1000; #4814, CST), rabbit anti-phospho-IκB-α monoclonal antibody (1:1000; #2859, CST), rabbit anti-iNOS monoclonal antibody (1:800; #13120, CST), mouse anti-PPAR-γ monoclonal antibody (1:1000; ab41928, Abcam), rabbit anti-ZO-1 polyclonal antibody (1:1000; 61–7300, Thermo Fisher Scientific), rabbit anti-occludin monoclonal antibody (1:1000; ab216327, Abcam), rabbit anti-claudin-1 polyclonal antibody (1:1000; 13050-1-AP, Proteintech), mouse anti-β-actin monoclonal antibody (1:5000; TA-09, Zsbio, China). The secondary antibodies used were goat anti-rabbit IgG (1:8000; ZB-2301, Zsbio, China) and goat anti-mouse IgG (1:8000; ZB-2305, Zsbio, China). The membranes were washed three times in TBST for 10 min each and incubated with species-specific anti-mouse/anti-rabbit peroxidase-conjugated secondary antibody for 1.5 h (ab6728, Abcam; ab6721, Abcam). Proteins were visualized using an enhanced chemiluminescence substrate (WP20005, Thermo Fisher Scientific). The densitometry of protein bands was quantified using ImageJ software.

### Transmission electron microscopy *analysis*

To examine the protective effect of HS on the colitis tissue, six animals from each group were sacrificed using cervical dislocation. The colons were collected, fixed immediately in 3.3% glutaraldehyde (4°C) for 24 h, washed in 0.1 M PBS, fixed in 1% osmium tetroxide for 3 h at 4°C, washed in 0.1 M PBS, dehydrated in an ascending alcohol series, soaked in propylene oxide, embedded in Epon 812 (45345, Sigma), sectioned on an ultramicrotome (LKB-V, Pharmacia, Sweden), stained with uranyl acetate and lead citrate, and examined with a transmission electron microscope (JEM-1400PLSER, JEOL, Japan).

### 16S rRNA gene surveys of fecal samples and pyrosequencing analysis

Fecal samples were collected before (at the end of day 10) and after DSS treatment (at the end of day 17). The samples were frozen in liquid nitrogen. DNA was isolated from fecal samples, amplified for the V4 region of the 16S rRNA gene, and sequenced using an Illumina HiSeq2500 instrument (Illumina, USA). The reads were processed and analyzed using QIIME (http://qiime.org/).

### Metagenomic sequencing

Fecal samples were collected from mice before (at the end of day 10) and after (at the end of day 17, before being sacrificed) drinking DSS water, which was frozen or refrigerated immediately after stool production. All samples were processed using the same pipeline in one laboratory (BGI Co., Ltd., China). Fecal DNA was isolated, and metagenomic shotgun sequencing was performed using the Illumina HiSeq, generating an average of 5.95 Gbp clean data per sample. The identified genes were compiled into a non-redundant catalog of 1,557,524 genes.

### Real-time quantitative polymerase chain reaction analysis

RNA from colonic tissue was isolated using Tri-reagent (Cwbio, China) according to the manufacturer’s instructions. RNA quantification was performed using NanoDrop ND-100 (Thermo Fisher Scientific). A reverse transcriptase reaction was performed to prepare complementary DNA (cDNA) using the EasyQuick RT MasterMix (Cwbio, China). A 1-µL volume of cDNA was used as a template for each real-time quantitative polymerase chain reaction (PCR) in a total reaction volume of 20 µL. Primers were designed online using NCBI and were synthesized by Shanghai Biotech. Real-time quantitative PCR was performed using SYBR Green (Q111-02,Vazyme Biotech Co., Ltd., China) on a sequence detection system (ABI Prism 7500, Applied Biosystems, USA). Data were analyzed using the comparative Ct method. The transcript level of the target gene was normalized to the mRNA level of the housekeeping gene *Gapdh*. The following primers were used for real-time quantitative PCR:

- *Nos2*: forward, 5′-GTTCTCAGCCCAACAATACAAGA-3′; reverse, 5′-GTGGACGGGTCGATGTCAC-3′
- *Gapdh*: forward, 5′-TGTAGACCATGTAGTTGAGGTCA-3′; reverse, 5′-AGGTCGGTGTGAACGGATTTG-3′

### SCFAs measurement

Cecal content was collected after DSS treatment (at the end of day 17). Fecal samples were collected before (at the end of day 10) and after DSS treatment (at the end of day 17). Samples were weighed into 1.5-mL tubes, crushed, and homogenized in 800 µL of distilled water. Subsequently, the tubes were vortexed for 1 min and centrifuged at 12,000 rpm for 20 min at 4°C. A 400-µL supernatant was absorbed to a new 1.5-mL centrifuge tube, to which 100 µL of 50% H_2_SO_4_ and 500 µL of diluted internal standard solution (50 µg/mL dimethyl pentanoic acid, dissolved in ethyl ether) was added. The tubes were vortexed for 1 min and centrifuged at 12,000 rpm for 20 min at 4°C. The samples were then placed in a refrigerator at 4°C for 30 min. The supernatant was collected in microtubes, and 2 µL was injected into the GC–MS (7010 B, Agilent Technologies, USA) under the following conditions: injector temperature, 250°C; initial oven temperature, 100°C; temperature increase of 5°C/min to 160°C, then 40°C/min to 240°C, and held for 10 min at the final temperature. Helium was used as the carrier gas at a rate of 1 mL/min. Finally, the resulting chromatograms were processed using ChemStation software (Agilent). Acetate, propionate, *n*-butyrate, *iso*-butyrate, *n*-valerate, and *iso*-valerate were quantified with appropriate calibration curves obtained using internal standard quantitation.

### Analysis of PMDZ dye retention in the colon

For the detection of hypoxia, mice were treated with 60 mg/kg of pimonidazole HCl (HP1-200Kit; Hypoxyprobe, USA) 30 min prior to sacrifice. Colon samples were fixed in 4% paraformaldehyde, and paraffin-embedded tissues were probed with mouse anti-pimonidazole monoclonal antibody (1:100; Hypoxyprobe) and then stained with Cy-3-conjugated goat anti-mouse antibody (1:50; SA00009-1, Proteintech), and then counterstained with DAPI using anti-fading mountant(S2110, Solarbio, China). Representative images were obtained using a Nikon fluorescent microscope (TE2000, Nikon, Tokyo, Japan).

### Scanning electron microscopy analysis

The colon specimens were fixed in 3.3% glutaraldehyde for 24 h at 4°C, washed in 0.1 M PBS, post-fixed in 1% osmium tetroxide for 3 h at 4°C, washed in 0.1 M PBS, dehydrated in a graded alcohol series, placed into isoamyl acetate, dried with liquid CO_2_ under pressure with a critical point dryer (Bio-Rad E 3000; Bio-Rad, UK), covered with gold particles (Bio-Rad SC502; Bio-Rad, UK), and observed using a JSM-6610LV scanning electron microscope (JEOL, Japan).

### Goblet cell staining

For goblet cell staining, distal colon tissues were fixed in Carnoy’s fixative and cut into 3-μm paraffin slices. After dewaxing and rehydration, the slices were stained with Alcian blue solution (1% w/v Alcian blue, 8GX in 3% v/v acetic acid, pH 2.5) (Solarbio, China) for 30 min. The slices were then washed with deionized water (dH_2_O) for 2 min. The nuclei were stained with 0.1% nuclear solid red (Solarbio, China) for 5 min and washed in dH_2_O for 1 min. The slices were then dehydrated, hyalinized, and sealed with neutral gum.

### Immunofluorescence staining

For MUC2 detection, mouse colons were fixed without flushing the luminal content with Carnoy’s fixative, embedded in paraffin, and sectioned at 3 μm. Following dewaxing and rehydration, slices were incubated for 20 min at 100°C in citrate buffer pH 6 (Solarbio, China) in a steamer for antigen retrieval. Slices were blocked for 1.5 h at room temperature in 5% BSA. Staining was performed with rabbit anti-MUC2 polyclonal antibody (1:1000; 27675-1-AP, Proteintech) and incubated overnight at 4°C. The secondary antibody was detected using an FITC-conjugated goat anti-rabbit secondary antibody (1:50; SA00003-2, Proteintech) and incubated for 1 h at room temperature. DAPI was incubated at room temperature for 10 min to stain the nuclei. The coverslips were mounted with an antifade mounting medium (Solarbio, China).

### Fluorescein isothiocyanate–dextran assay

An in vivo assay of intestinal permeability was performed using FITC–dextran, as described previously.^58^ Briefly, mice were deprived of food and water for 4 h and then orally gavaged with 0.6 mg/g body weight of 4 kDa FITC–dextran (FD4; Sigma). Blood was collected retro-orbitally after 4 h, and FITC fluorescence intensity was measured in the serum samples (excitation, 488 nm; emission, 520 nm). FITC–dextran concentrations were determined using a standard curve generated by performing serial dilution of FITC–dextran in mouse serum.

### Statistical analysis

All statistical analyses were performed using SPSS version 19.0, for Windows (SPSS Inc., Chicago, IL, USA). The normality of continuous variables was assessed using the Shapiro-Wilk test. Continuous variables of normality distributions were expressed as mean ±standard deviation (SD), and one-way ANOVA with LSD multiple comparison post hoc test was used to compare the differences between groups. Non-normally distributed data (e.g., α-diversity estimation of microbial community) were described with the median and interquartile range (IQR) and analyzed with the Kruskal-Wallis test. Statistical significance was set at *P* < .05.

## Supplementary Material

Supplemental Material

## Data Availability

All data generated in this study are included in this article and supplementary information or are available from the corresponding author on reasonable request. https://figshare.com/articles/figure/Microbial_hydrogen_economy_alleviates_colitis_by_reprogramming_colonocyte_metabolism_and_reinforcing_intestinal_barrier/15130824
