# Supplementary material for: Microbial hydrogen economy alleviates colitis by reprogramming colonocyte metabolism and reinforcing intestinal barrier
Source: Gut Microbes. 2022 Jan 13;14(1):2013764. doi: 10.1080/19490976.2021.2013764 (PMC8759589; doi:10.1080/19490976.2021.2013764)
Supplement: Supplemental Material [file KGMI_A_2013764_SM6258.zip › supplementary/supplement material.docx]

***Supplemental Materials and Methods***

**16S Ribosomal RNA (rRNA) pyrosequencing**

Fecal samples were respectively collected from mice before (at the end of day10, 10d) and after (at the end of day17, 17d, before being killed) drinking DSS water, snap-frozen in liquid N2 and shipped to the laboratory on dry ice. The microbial community DNA was extracted using MagPure Stool DNA KF kit B(Magen, China)following the manufacturer's instructions. DNA was quantified with a Qubit Fluorometer by using Qubit dsDNA BR Assay kit (Invitrogen, USA) and the quality was checked by running aliquot on 1% agarose gel. Variable regions V4 of bacterial 16S rRNA gene was amplified with degenerate PCR primers, 515F (5’-GTGCCAGCMGCCGCGGTAA-3’) and 806R(5’-GGACTACHVGGGTWTCTAAT-3’). Both forward and reverse primers were tagged with Illumina adapter, pad and linker sequences. PCR enrichment was performed in a 50 μL reaction containing 30ng template, fusion PCR primer and PCR master mix. PCR cycling conditions were as follows: 95°C for 3 minutes, 30 cycles of 95°C for 45 seconds, 56°C for 45 seconds, 72°C for 45 seconds and final extension for 10minutes at 72°C for 10 minutes. The PCR products were purified using Agencourt AMPure XP beads and eluted in Elution buffer. Libraries were qualified by the Agilent Technologies 2100 bioanalyzer. The validated libraries were used for sequencing on Illumina HiSeq 2500 platform (BGI, Shenzhen, China) following the standard pipelines of Illumina, and generating 2 × 250 bp paired-end reads.

Raw reads were filtered to remove adaptors and low-quality and ambiguous bases, and then paired-end reads were added to tags by the Fast Length Adjustment of Short reads program (FLASH, v1.2.11) to get the tags.^1^ The tags were clustered into OTUs with a cutoff value of 97% using UPARSE software (v7 .0.1090) and chimera sequences were compared with the Gold database using UCHIME (v4.2.40) to detect.^2^ ^3^Then, OTU representative sequences were taxonomically classified using Ribosomal Database Project (RDP) Classifier v.2.2 with a minimum confidence threshold of 0.6, and trained on the Greengenes database v201305 by QIIME v1.8.0.^4^ The USEARCH_global was used to compare all Tags back to OTU to get the OTU abundance statistics table of each sample. Alpha and beta diversity were estimated by MOTHUR (v1.31.2) and QIIME (v1.8.0) at the OTU level, respectively.^4-6^ Barplot and heatmap of different classification levels was plotted with R package v3.4.1 and R package “gplots”, respectively. Nonmetric multidimensional scaling ordination (NMDS) was performed by R package. LEfSe cluster or LDA analysis was conducted by LEfSe. Significant Species were determined by R(v3.4.1) based on Wilcox-test or Kruskal-Test.

**Metagenomic sequencing.** Fecal samples were respectively collected from mice before (at the end of day10, 10d) and after (at the end of day17, 17d, before being killed) drinking DSS water，which was frozen or refrigerated immediately after stool production. After DNA extraction, 1µg genomic DNA was randomly fragmented by Covaris, followed by purification by Axy Prep Mag PCR clean up kit. The fragmented DNA was selected by Agencourt AMPure XP Medium kit to an average size of 200-400bp. The fragments were end repaired by End Repair Mix and purified afterwards. The repaired DNAs were combined with A-Tailing Mix, then the Illumina adaptors were ligated to the Adenylate 3’Ends DNA and followed by purification. The products were selected based on the insert size. Several rounds of PCR amplification with PCR Primer Cocktail and PCR Master Mix were performed to enrich the Adapter-ligated DNA fragments. After purification, the library was qualified by the Agilent 2100 bioanalyzer (Agilent, USA) and ABI StepOnePlus Realtime PCR System. Finally, the qualified libraries were sequenced on illumina Hiseq platform (BGI-Shenzhen, china). All the raw data were trimmed by SOAPnuke v.1.5.2.^7^ The trimmed reads were mapped to the host genome using SOAP2 software to identify and remove host originated reads (only for samples of host resources).^8^ High-quality reads were *de novo* assembled using IDBA-UD software. Assembled contigs with length less than 300 bp were discarded in the following analysis.^9^ Genes were predicted over contigs by using MetaGeneMarker (2.10).^10^ Redundant genes were removed using CD-HIT with identity cutoff 95%.^11^ To generate the taxonomic information, the protein sequences of genes were aligned against the NR database using DIAMOND with an E value cutoff of 1e−5.^12^ Based on the MEGAN LCA algorithm, the taxonomic annotation was assigned.^13^ To obtain functional information, the protein sequences were aligned against the eggNOG database (2015-10), CAZy database (2017-09), COG database (2014-11), Swiss-prot database (2017-07), the KEGG database (89.1) and CARD database (4.0) by DIAMOND with an E value cutoff of 1e−5.^12^ To generate the taxonomic and functional abundance profiles, the reads were aligned to the genes using Botwie2 with the default setting. Based on the abundance profiles, the features (Genera, Phyla and KOs) with significantly differential abundances across groups were determined using Wilcoxon’s rank sum test.^14, 15^ *P*-values for multiple testing were corrected using the BH method with corrected *P*-values<0.05 were considered significant. Differentially enriched KEGG pathways were identified according to reporter scores.^16^ An absolute value of reporter score of 1.65 or higher was used as the detection threshold for significance. The alpha diversity was quantified by the Shannon index using the relative abundance profiles at gene, genus and KO levels with R package. The beta diversity was calculated using Bray-Curtis distance or Jensen-Shannon Divergence distance. Principal component analysis (PCA) was plotted with R package “ade4”. Principal Coordinate Analysis (PCoA) was performed by R package VEGAN.

**References**

1. Magoc T, Salzberg SL. FLASH: fast length adjustment of short reads to improve genome assemblies. Bioinformatics 2011; 27:2957-2963. doi:10.1093/bioinformatics/btr507. PMID:21903629.

2. Edgar RC. UPARSE: highly accurate OTU sequences from microbial amplicon reads. Nat Methods 2013; 10:996-998. doi:10.1038/nmeth.2604. PMID:23955772.

3. Edgar RC, Haas BJ, Clemente JC, Quince C, Knight R. UCHIME improves sensitivity and speed of chimera detection. Bioinformatics 2011; 27:2194-2200. doi:10.1093/bioinformatics/btr381. PMID:21700674.

4. Caporaso JG, Kuczynski J, Stombaugh J, Bittinger K, Bushman FD, Costello EK, Fierer N, Pena AG, Goodrich JK, Gordon JI, et al. QIIME allows analysis of high-throughput community sequencing data. Nat Methods 2010; 7:335-336. doi:10.1038/nmeth.f.303. PMID:20383131.

5. Edgar RC. Search and clustering orders of magnitude faster than BLAST. Bioinformatics 2010; 26:2460-2461. doi:10.1093/bioinformatics/btq461. PMID:20709691.

6. Schloss PD, Westcott SL, Ryabin T, Hall JR, Hartmann M, Hollister EB, Lesniewski RA, Oakley BB, Parks DH, Robinson CJ, et al. Introducing mothur: open-source, platform-independent, community-supported software for describing and comparing microbial communities. Appl Environ Microbiol 2009; 75:7537-7541. doi:10.1128/AEM.01541-09. PMID:19801464.

7. Chen Y, Chen Y, Shi C, Huang Z, Zhang Y, Li S, Li Y, Ye J, Yu C, Li Z, et al. SOAPnuke: a MapReduce acceleration-supported software for integrated quality control and preprocessing of high-throughput sequencing data. Gigascience 2018; 7:1-6. doi:10.1093/gigascience/gix120. PMID:29220494.

8. Li R, Yu C, Li Y, Lam TW, Yiu SM, Kristiansen K, Wang J. SOAP2: an improved ultrafast tool for short read alignment. Bioinformatics 2009; 25:1966-1967. doi:10.1093/bioinformatics/btp336. PMID:19497933.

9. Peng Y, Leung HC, Yiu SM, Chin FY. IDBA-UD: a de novo assembler for single-cell and metagenomic sequencing data with highly uneven depth. Bioinformatics 2012; 28:1420-1428. doi:10.1093/bioinformatics/bts174. PMID:22495754.

10. Zhu W, Lomsadze A, Borodovsky M. Ab initio gene identification in metagenomic sequences. Nucleic Acids Res 2010; 38:e132. doi:10.1093/nar/gkq275. PMID:20403810.

11. Fu L, Niu B, Zhu Z, Wu S, Li W. CD-HIT: accelerated for clustering the next-generation sequencing data. Bioinformatics 2012; 28:3150-3152. doi:10.1093/bioinformatics/bts565. PMID:23060610.

12. Buchfink B, Xie C, Huson DH. Fast and sensitive protein alignment using DIAMOND. Nat Methods 2015; 12:59-60. doi:10.1038/nmeth.3176. PMID:25402007.

13. Huson DH, Auch AF, Qi J, Schuster SC. MEGAN analysis of metagenomic data. Genome Res 2007; 17:377-386. doi:10.1101/gr.5969107. PMID:17255551.

14. Langmead B, Salzberg SL. Fast gapped-read alignment with Bowtie 2. Nat Methods 2012; 9:357-359. doi:10.1038/nmeth.1923. PMID:22388286.

15. Matsouaka RA, Singhal AB, Betensky RA. An optimal Wilcoxon-Mann-Whitney test of mortality and a continuous outcome. Stat Methods Med Res 2018; 27:2384-2400. doi:10.1177/0962280216680524. PMID:27920364.

16. Patil KR, Nielsen J. Uncovering transcriptional regulation of metabolism by using metabolic network topology. Proc Natl Acad Sci U S A 2005; 102:2685-2689. doi:10.1073/pnas.0406811102. PMID:15710883.
