# Supplementary figures and images for: Microbial hydrogen economy alleviates colitis by reprogramming colonocyte metabolism and reinforcing intestinal barrier
Source: Gut Microbes. 2022 Jan 13;14(1):2013764. doi: 10.1080/19490976.2021.2013764 (PMC8759589; doi:10.1080/19490976.2021.2013764)

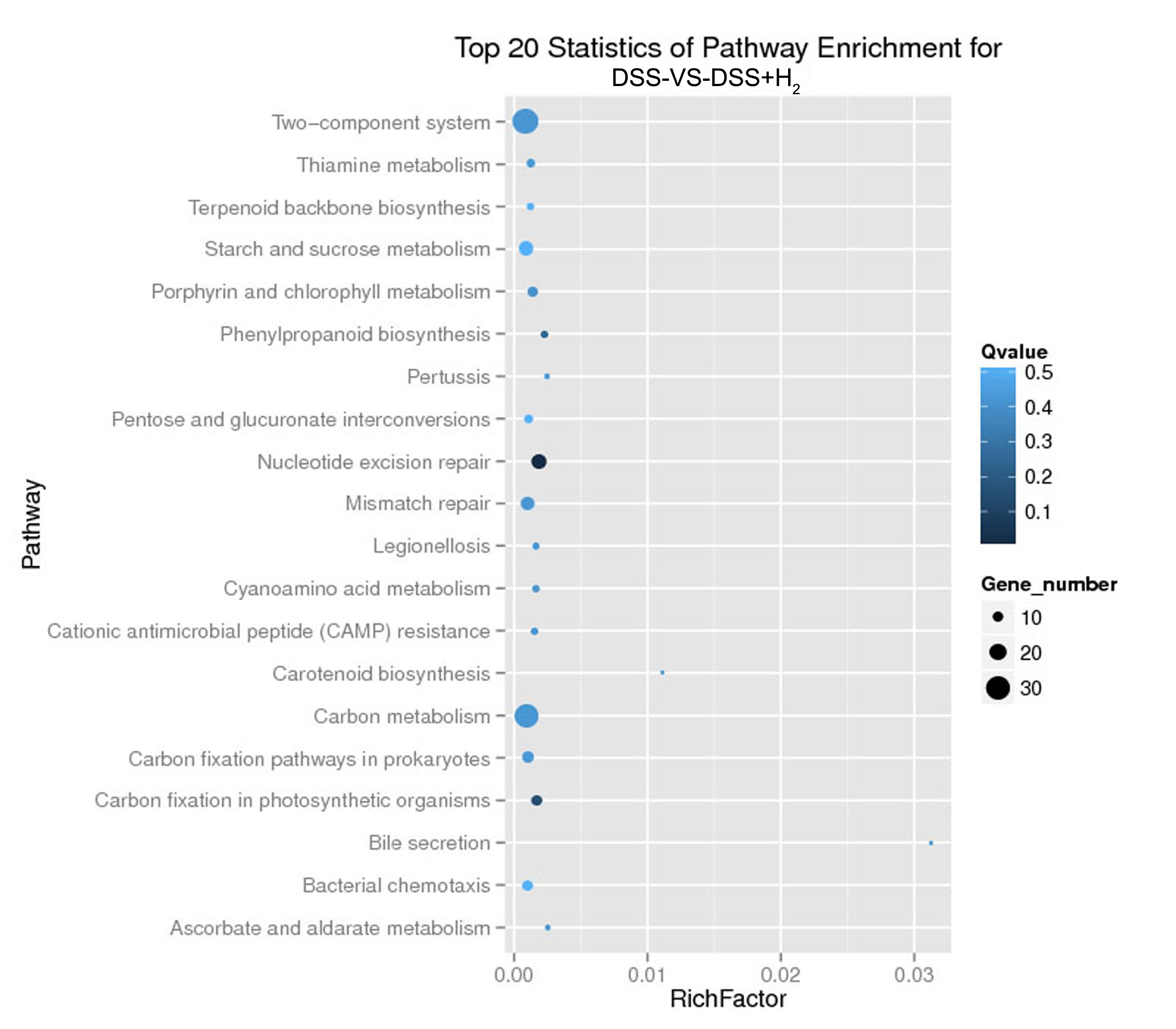

Supplement: Supplemental Material [file KGMI_A_2013764_SM6258.zip › supplementary/Figure S3.jpg]

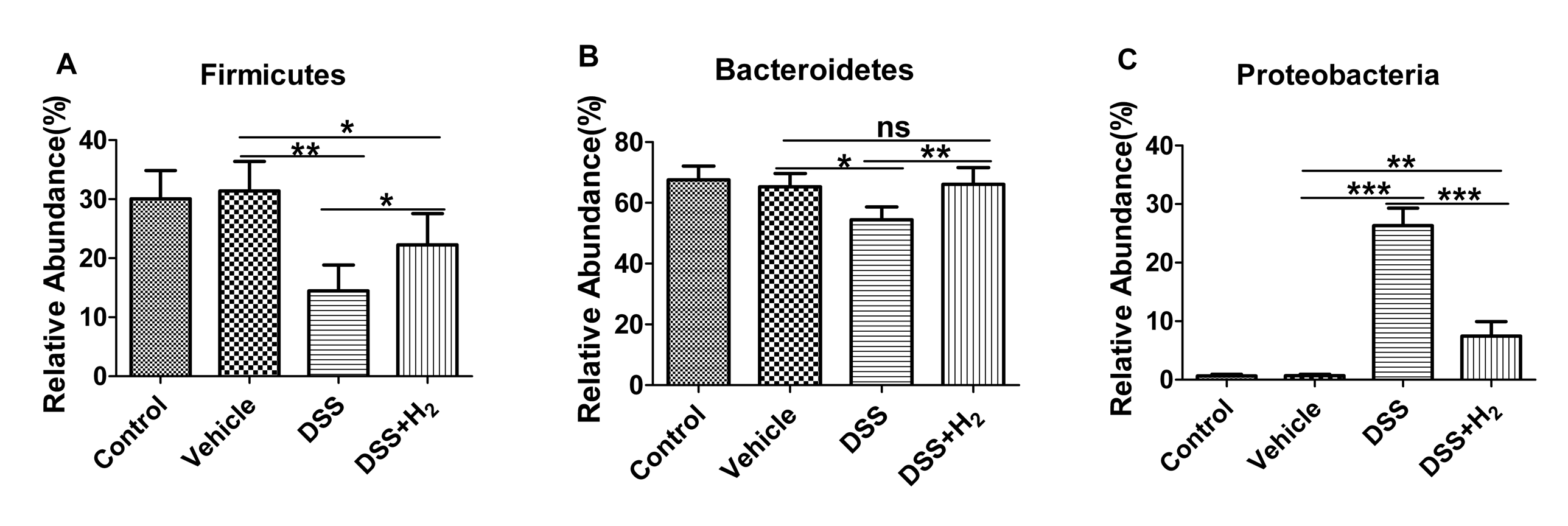

Supplement: Supplemental Material [file KGMI_A_2013764_SM6258.zip › supplementary/revised Figure S1.tif]

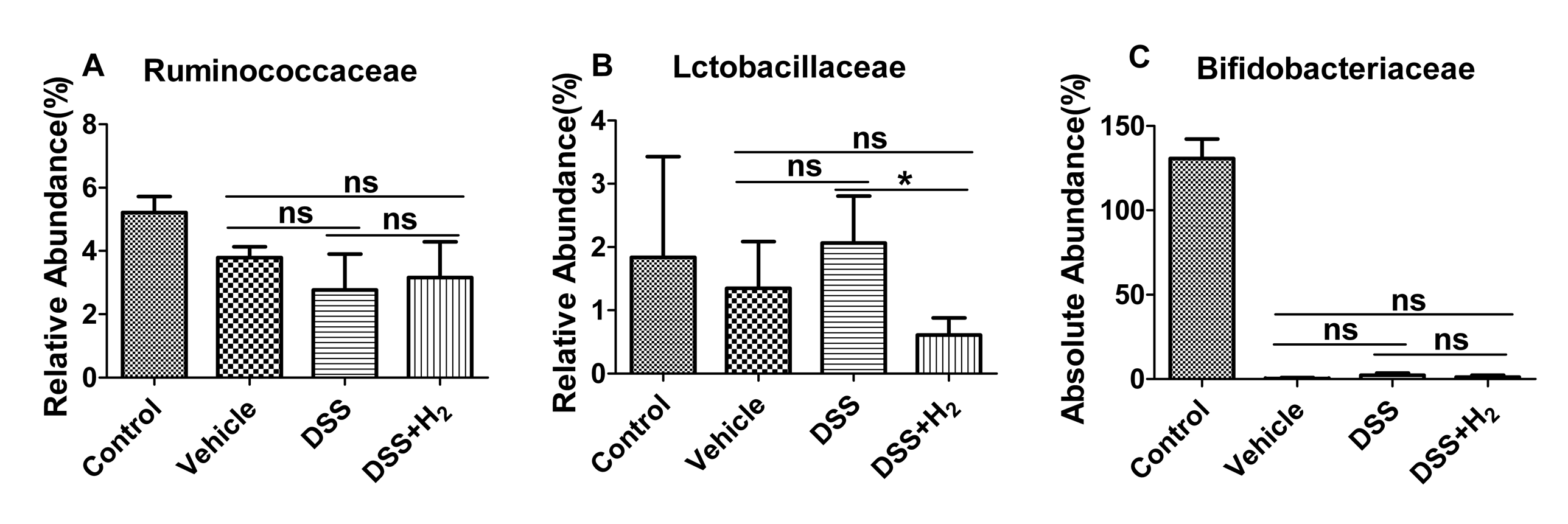

Supplement: Supplemental Material [file KGMI_A_2013764_SM6258.zip › supplementary/revised Figure S2.tif]
